# Early outcome after the use of the triceps fascia flap in interposition elbow arthroplasty: a novel method in the treatment of post-traumatic elbow stiffness

**DOI:** 10.1051/sicotj/2020006

**Published:** 2020-05-05

**Authors:** Emmanuel C. Iyidobi, Cajetan U. Nwadinigwe, Remigus T. Ekwunife, Udo E. Anyaehie, Lasebikan A. Omolade, Osita Ede, Valentine Okeke

**Affiliations:** 1 Department of Orthopaedics and Trauma, National Orthopaedic Hospital HA7 4LP Enugu Nigeria

**Keywords:** Elbow, Interposition arthroplasty, Triceps fascia, Post-traumatic stiffness

## Abstract

*Background*: Post-traumatic elbow stiffness (PTES) results in severe interference with the activities of daily living (ADL), affecting mainly young people. Total elbow arthroplasty (TEA) is relatively contraindicated in the young patient and arthrodesis is poorly tolerated. Interposition elbow arthroplasty (IEA) improves the range of motion (ROM) buying time for future reconstructive surgery. While the fascia lata remains the most common material used in IEA, the triceps fascia is a native vascularized tissue, and it does not require a separate incision to harvest. To our knowledge, there are no published studies on the use of this technique of IEA. *Method*: Sixteen patients with post-traumatic elbow stiffness had IEA with the triceps fascia between January 2009 and January 2017. The ROM was assessed pre-operatively and post-operatively at the 6th and the 24th week. The researchers also evaluated the functional outcome with the Mayo Elbow Performance Score (MEPS) at the 24th week. The data were analysed with the software IBM SPSS Version 20. *Results*: Nine males and seven females had IEA with the triceps fascia. The mean age of the subjects was 22.8 years (SD = 6.39). The median duration of the stiffness was eight months (range: 2–168 months). Fall was the most frequent cause of post-traumatic elbow stiffness, and the non-dominant side was more frequently involved. Fourteen patients had an intervention at the native bone setters before presentation to the hospital. The mean elbow ROM increased from 16.4° pre-operatively to 97.2° at the 24th week (*p* < 0.001), while the mean MEPS improved from 42.5° pre-operatively to 81.2° post-operatively (*p* < 0.001). *Conclusion*: The triceps fascia flap provides an excellent alternative to the fascia lata for IEA without the complications of the donor site morbidity.

## Introduction

The elbow joint comprises three articulations and serves to position the hand in space for the ADLs. Because of the propensity of the elbow to develop stiffness after injury [1], it is regarded as the most unforgiving joint in the body. The elbow has a functional ROM of 100° of flexion-extension and 100° of pronation–supination, and any greater loss is likely to be disabling [1, 2]. The loss of 50% of elbow function equals a loss of 80% of the total upper extremity function [3]. Such patients find it difficult to reach the hair, mouth or buttocks for hygiene.

Elbow stiffness is a relatively rare orthopaedic condition. According to literature, the incidence of post-traumatic elbow stiffness is about 5% [1]. Post-traumatic arthrosis appears to be the leading cause in most literature [2, 3]. Other less common causes include rheumatoid arthritis and infection [2, 4]. Regardless of the initiating pathology, the result is a stiff elbow that compromises the patient’s ability to carry out the ADLs.

Post-traumatic elbow stiffness (PTES) commonly complicates elbow injury in our environment. Patients often consult the native bonesetters, who splint the elbow usually in extension for a prolonged period. Such patients often come down with stiffness in addition to the primary elbow pathology which frequently is uncorrected by the splint. Most of such injuries include supracondylar fractures in children and elbow fracture-dislocations in adults.

Total elbow arthroplasty (TEA), resection arthroplasty, arthrodesis, and interposition elbow arthroplasty (IEA) are options for treating elbow stiffness from varying aetiologies [3–6]. However, TEA is relatively contraindicated in the young patient because of early loosening of the prosthesis [5]. The active young patient frequently exceeds the 5-kg weight limit of the prosthesis. Resection arthroplasty and arthrodesis are also poorly tolerated by the young individual because of extreme instability and extreme limitation in the ROM, respectively, associated with the procedures [7].

Interposition elbow arthroplasty uses natural or synthetic materials to separate the humero-ulnararticular surfaces. Materials used include the Fascia lata, Achilles tendon allograft, skin graft, Anconeus muscle and gelfoam [5, 8, 9]. The fascia lata is the commonest material used in most studies [5, 9]. However, problems associated with the use of the fascia lata include a separate skin incision for harvesting, the risks of a muscle hernia, lesion of the lateral femoral cutaneous nerve and donor site pain and infection.

The triceps fascia is the deep fascia of the forearm overlying the triceps muscle. This fascia does not require a separate incision to harvest and retains its vascular supply. The vascularity of the flap may enable it to survive for a prolonged period, which has been difficult to achieve with the use of other non-vascular materials. It is believed that the interposition material will eventually be reabsorbed with time and results in recurrence of the elbow stiffness. Hence, these potential advantages make the triceps fascia flap an attractive alternative in comparison to the other materials for the interposition arthroplasty.

## Materials and methods

This study was a prospective interventional study conducted at National Orthopaedic Hospital, Enugu (NOHE), in Nigeria between January 2009 and January 2017. NOHE is a regional centre for Orthopaedics and Trauma in the South-East region of Nigeria. The inclusion criteria were patients who presented with elbow stiffness secondary to elbow injury with less than the functional elbow ROM (100°), while patients with non-traumatic causes of elbow stiffness, patients with more than the elbow functional ROM and those that refuse to give consent were excluded from the study. Ethical approval was obtained from the Hospitals’ Ethics Committee and written informed consent was received from each subject before the study.

**Figure 1:**
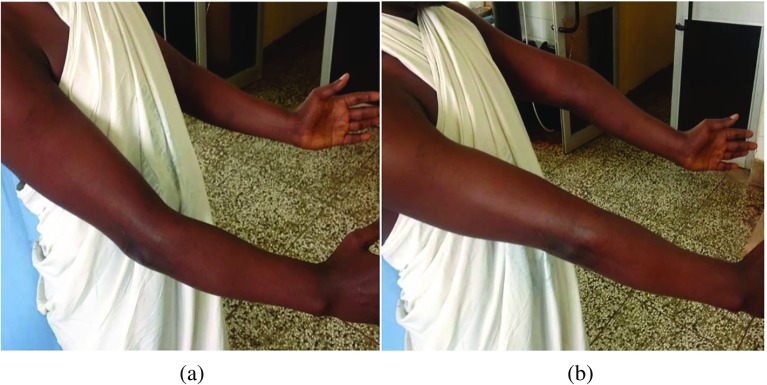
(a and b) Maximum flexion and extension in a patient with bilateral elbow stiffness.

**Figure 2:**
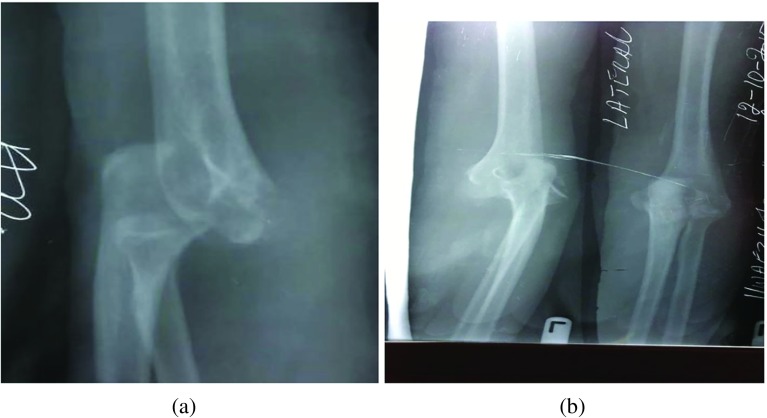
(a and b) The anterior–posterior and lateral X-rays of a patient showing unreduced elbow dislocation. Note the disruption of the radio-capitellar line in the lateral view.

We assessed the pre-operative ROM of the elbow with a goniometer (AO Education). We also evaluated the pre-operative Mayo Elbow Performance Scores (MEPS). This score has four components: Pain, Motion, Function and Stability. The maximum score is 100, and the total score is excellent if greater than 75, fair if between 60 and 75 and poor if less than 60 [6]. The nerve function of the limb was assessed for neuropathy. We evaluated the pre-operative AP and lateral radiographs of the affected elbow. Four surgeons did all the operations (Figures 1 and 2).

**Figure 3:**
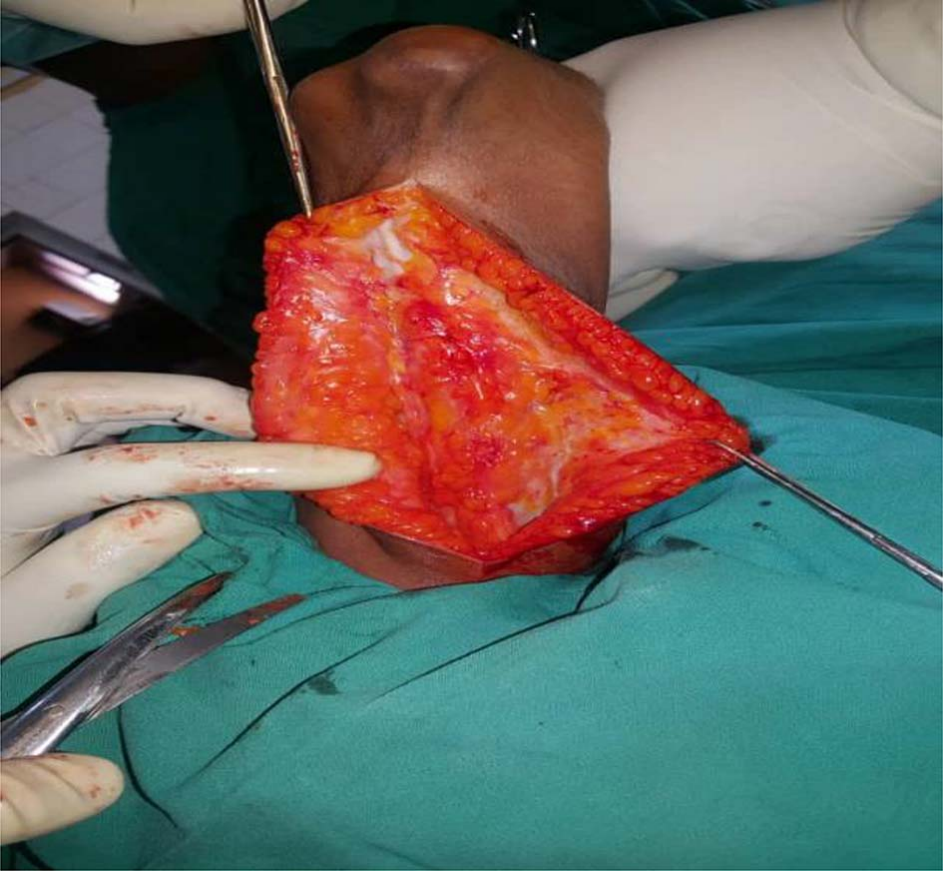
The proximal part of the skin and subcutaneous flaps to expose the triceps muscle and its fascia.

The patient was positioned supine with the arm across the chest to allow intra-operative flexion and extension. A high arm tourniquet was applied in all the cases. A posterior skin incision was made in the distal half of the arm and carried medially around the tip of the olecranon to the proximal third of the forearm. A medial and the lateral subcutaneous flaps were raised to reveal the deep fascia covering the triceps muscle (see Figure 3).

**Figure 4:**
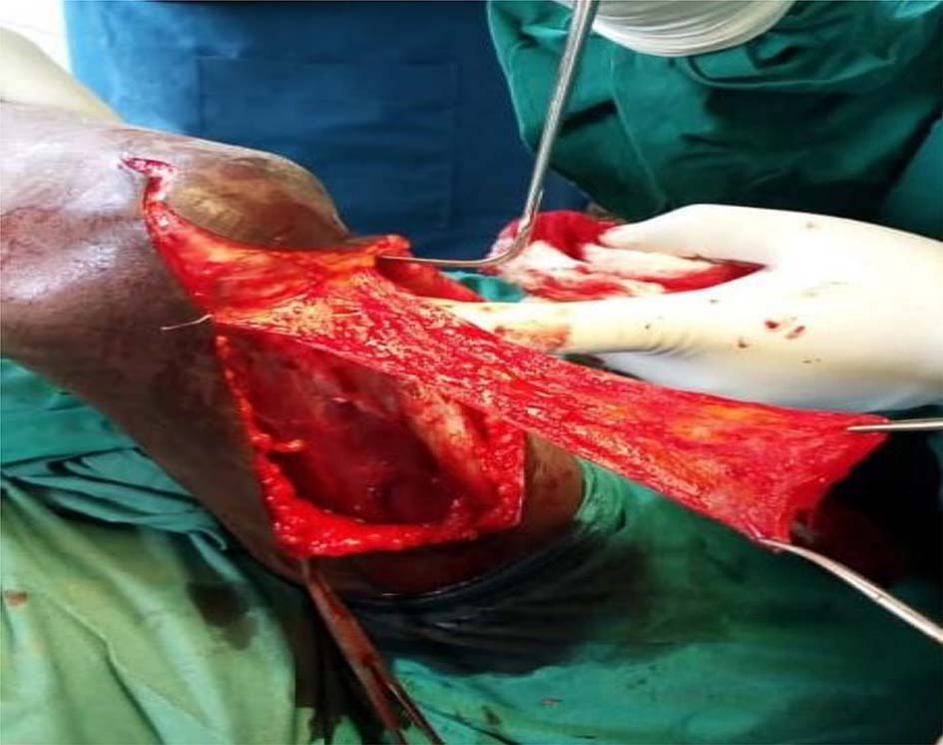
The triceps fascia raised with the base distal. Note the distal continuation of the skin incision used to gain access to the joint cavity.

The ulnar nerve was identified and protected by releasing it from the cubital tunnel and tying a vascular loop loosely around it. We dissected out the fascial flap with the base distal, i.e. towards the elbow, being careful not to cause multiple buttonholes in the flap (see Figure 4). The underlying exposed triceps aponeurosis is now split in the midline down to the bone and elevated subperiosteally off the humerusin a medial and lateral directions.

**Figure 5:**
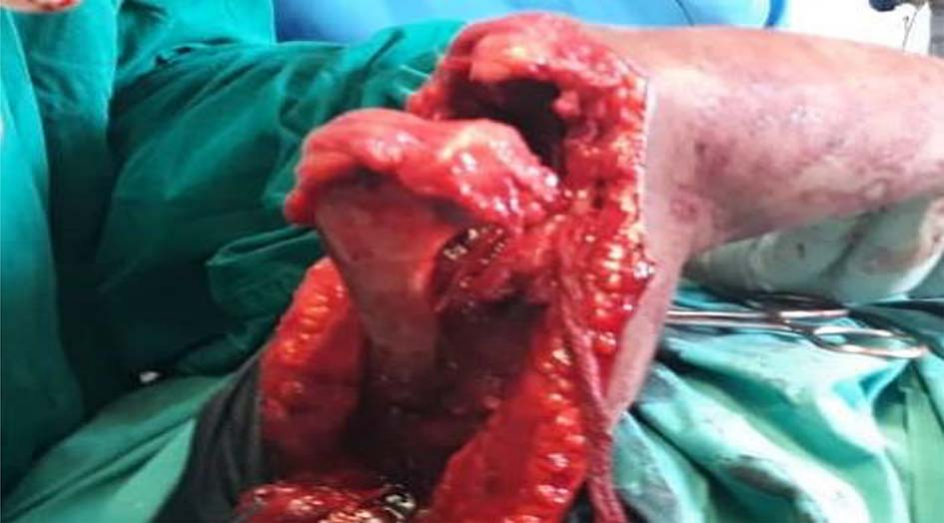
The triceps fascia is seen covering the humeral articular surface.

A posterior capsulectomy was performed, and the elbow flexed to expose the joint cavity. The two elbows in the fixed extension position required a V-Y triceplasty to be able to gain flexion. Once inside, we cleared the cavity of pannus and excised any bony block. This procedure revealed the anterior capsule and enabled the surgeon to complete the capsulectomy to gain maximal ROM. The intra-operative ROM is now assessed. If satisfactory, the triceps fascia flap is then tunnelled anteriorly and wrapped over the articular surface of the distal humerus (see Figure 5). It is secured to the cut edges of the excised capsule with non-absorbable sutures (Ethilon 0). The surgeon assessed the elbow for valgus and varus stability. If unstable, we plicate the collateral ligaments or directly suture repair them if they were torn.

The joint is now irrigated and reduced. The wound is closed over a suction drain, and a removable POP back slab was applied with the elbow in 90° of flexion and mid-prone. The drain was removed on the 2nd postoperative day, and the patients commenced on active assisted ROM exercises, removing the back slab for each exercise session. The exercise is done three times a day. The patients routinely received tablet Indomethacin 25 mg thrice daily to prevent heterotopic ossification. Patients were prohibited from massaging the elbow and lifting weights that exceed 1-kg for six months after the surgery. Sutures were removed in the second week, and the surgeon assessed the post-operative ROM at the 6th and the 24th week. The MEPS were also evaluated at the 24th week. The POP back slab was changed to a removable brace at the 6th week and continued active ROM exercise was encouraged.

The data were analysed with the software IBM SPSS Version 20. Categorical variables were represented as frequencies while continuous variables were expressed as means (SD) if the distributions were normal or as medians (minimum–maximum) if the distribution is not normal. The distribution of variables was analysed with the Shapiro–Wilks test. A one-way repeated measure analysis of variance (ANOVA) was used to assess the difference in the ROM before and after surgery, while the Wilcoxon signed-rank test was used to evaluate the difference in the MEPS scores pre-operatively and post-operatively. A *p*-value of less than 0.05 was considered significant.

## Results

Interposition elbow arthroplasty was performed with the Triceps fascia flap on 16 patients with PTES. Nine males (56.3%) and seven females (43.8%) were involved. The mean age of the subjects was 22.8 years (SD = 6.4). The median duration of the stiffness was eight months (2–168 months). Fall was the most frequent aetiology of PTES, accounting for nine cases (56.3%), and the most frequent pathology found on X-ray was unreduced elbow dislocation. All the subjects were right-handed, and the left side was affected more than the right (56.3% vs. 43.8%). Approximately 88% of subjects visited the native bonesetters. Only one case suffered postoperative radial nerve palsy which recovered entirely by the 6th week. Table 1 summarizes the characteristics of the subjects.

**Table 1:** Subject characteristics (*N* = 16).

Subject characteristics	*N*
Gender	
M	9
F	7
Age categories (years)	
10–20	5
21–30	9
31–40	2
Occupation	
Students	7
Applicants	5
Teachers	2
Trader	1
Artisan	1
Duration of stiffness (months)	
<12	10
13–24	4
>24	2
Aetiology	
Fall	9
Assault	4
Road traffic accident	3
Diagnosis	
Unreduced left elbow dislocation	9
Unreduced right elbow dislocation	4
Malunited right supracondylar fracture	3
Pre-hospital treatment	
Native bone setters	14
Private clinics	2

**Figure 6:**
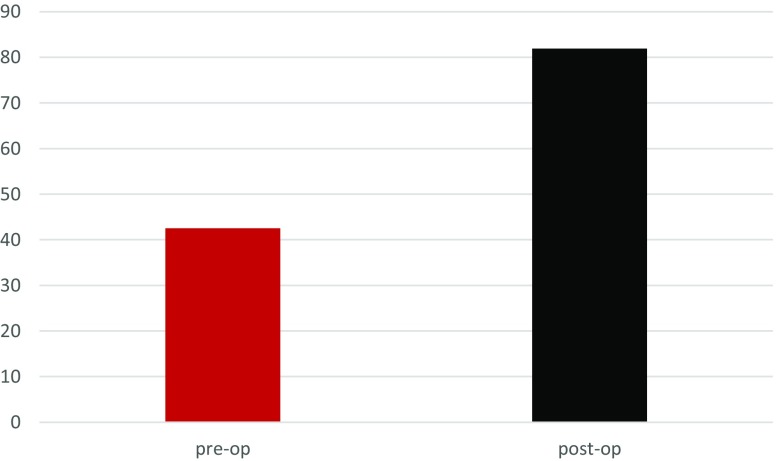
The preoperative and postoperative MEP Scores.

**Figure 7:**
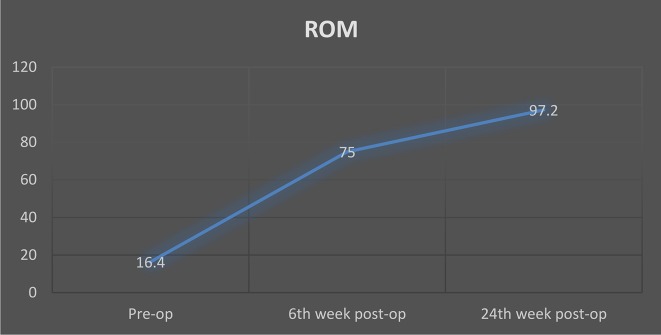
The trend in the ROM from the preoperative period to the assessment at the 24th week postoperative visit.

The mean pre-operative ROM of the elbow was 16.4° (range: 0°–40°). Two subjects had the elbow in fixed extension. We were able to get a mean ROM of 121.3° intra-operatively (range: 80°–140°), (*p* < 0.001). The average postoperative ROM was 75° (range: 35°–115°) at six weeks and 97.2° (range: 70°–120°) at 24 weeks. The mean pre-operative MEPS was 42.5 (SD = 8.9), while it increased to 81.9 (SD = 9.5) at the 24-week post-operative visit (*p* ≤ 0.001), see Table 2. Figure 6 is a bar chart illustrating the increased MEPS Scores, while Figure 7 illustrates the trend in the ROM over the study period.

**Table 2:** The mean values of the various components of the MEPS. Standard deviations are enclosed within brackets.

Components	Mean pre-operative score	Mean post-operative score
Pain	23.44 (10.44)	41.25 (6.71)
Range of motion	6.25 (3.42)	15.94 (2.02)
Function	5.94 (3.28)	17.19 (4.07)
Stability	6.88 (2.50)	6.86 (2.49)
Total	42.51	81.24

The results of the repeated measure ANOVA showed a significant time effect for the ROM before and after surgery at the 6th and the 24th post-operative week, Wilks Lambda = 0.03, *F* (2, 14) = 211.7, *p* ≤ 0.001, *η*^2^ = 0.97. Thus, we reject the null hypothesis. Follow-up comparisons indicated that each pairwise difference was significant, *p* ≤ 0.001. Therefore, the ROM improves with time in the subjects.

## Discussion

It has been shown in this study that the triceps fascia flap gives relatively good result in the early periods following interposition arthroplasty for posttraumatic elbow stiffness. All the subjects in this study were below 40 years; thus, they have a high ambition and activity level in everyday life. Most studies used the fascia lata and other materials as interposition materials [5, 8]. Apart from the complications of donor site morbidity and the theoretical risk of infection from an allograft, a significant concern is that of eventual resorption of the material with a recurrence of elbow stiffness [5]. It is possible that this may be because these tissues are not vascularized.

The triceps fascia flap retains its vascularity and may potentially last longer than the other materials, which is an essential consideration in our environment because widespread poverty and ineffective health insurance will make a future elbow arthroplasty unaffordable for many patients. Therefore, any surgery that will preclude a future arthroplasty will be a welcome development. Larson and Morrey have shown in their work with an average follow up of six years that the elbow still maintained reasonable mean ROM of 97° [6]. They used the Achilles tendon allograft, which is non-vascularized. We expect that the vascularized triceps fascia flap will outlive this allograft.

Elbow injuries in our subjects are unique since the native bonesetters usually complicate them before the patients present to the hospital. At this time, fibrosis and soft tissue contractures make the surgery extremely difficult. Most of the times, the primary pathology, usually an unreduced elbow dislocation, will still be present together with the attendant stiffness. The contracture of the triceps muscle means that V-Y triceplasties are often needed to achieve elbow flexion and joint reduction. The surgery may be more complicated than the ones for the straightforward pathologies without joint luxations encountered in other studies.

External fixators were used in some studies to stabilize the elbow while the soft tissues heal [3, 6, 7]. Larry and Morrey have shown no difference in the outcome between patients managed with and without an external fixator, even though they noted the bias in using the fixator for patients deemed to have an unstable elbow at the time of reconstruction [6]. We used removable POP back slab to provide initial stability for six weeks postoperatively and change to a removable brace, which the subject wears for the next 24 weeks. The slab and the brace are removed for active ROM exercises. We did not observe any disabling post-operative instability in the subjects.

The ROM improved over time in our study. It is expected that the ROM will improve as pain decreases. Even though the mean ROM at the 24th week is a little less than the recommended 100°, the majority of the subjects were able to reach their hair, mouth and buttocks and were quite happy with the results. The mean MEPS was 81.9, which was higher than the 75 from the study of Jaiswal et al. [4], but less than the 100 observed by Rollo et al. [5]. The former utilized the fascia lata which was sutured over the head of the humerus and had an average follow up of six months like in our study, while the latter used allogeneic fascia lata which was sutured over the distal humerus and olecranon. Running sutures were applied to the edges of the graft, and transosseous sutures were used to fix the graft to both the humerus and olecranon. They had an average follow up of five years. It is possible that as the subjects become better adapted to the new way their reconstructed elbow functions, the MEPS may improve. However, the shortcoming of both studies is that they were case reports and findings may not be generalized to the larger population.

The only complication noted in this study was a case of postoperative radial nerve palsy, thought to be related to the tourniquet used during the surgery. The patient recovered full radial nerve function by the sixth-week postoperative visit. Of interest is the lack of donor site morbidities associated with the other methods of IEA, which is a considerable advantage of this new method. Another technique that is devoid of donor site morbidity in the treatment of elbow ankylosis is the use of the reverse lateral arm fascial flap described by Udo et al. in our hospital [10]. However, there has not been a follow-up study on patients treated with this method; hence, the results have yet to be validated.

## Conclusion

The triceps fascia flap has demonstrated a good outcome for interposition elbow arthroplasty in the short term. The retained vascularity and the lack of donor site morbidities make it an attractive choice for IEA.

## Study limitations

A longer follow-up is needed to study the long-term performance of this method. A multicentre study will also increase the sample size.
